# Anti-Voltage-Gated Potassium Channel Antibody Limbic Encephalitis Mimicking Paraneoplastic Syndrome

**DOI:** 10.51894/001c.144199

**Published:** 2025-09-30

**Authors:** Nahel Abugattas

**Affiliations:** 1 College of Osteopathic Medicine Michigan State University

## 55

### BACKGROUND

Limbic Encephalitis (LE) is a rare neurological inflammatory process affecting the hypothalamus and limbic system. It is characterized by subacute behavioral and mood changes, seizure, memory loss, and cognitive decline that may progress to dementia. While over 50% of LE cases are associated with underlying malignancies, a minority can be attributed to autoimmune etiologies such as Anti-Voltage-Gated Potassium Channel (VGKC) Encephalitis.

### CASE

We present the case of a 66 year old male initially hospitalized at a different facility for a new onset seizure with secondary intubation due to prolonged postictal state. Additional history revealed several months of mood swings, episodes of aggression, confusion, and memory loss. Patient was discharged to subacute rehabilitation, but presented to our facility 6 days later with altered mental status, gait dysfunction, lower extremity muscle weakness, and increased urinary frequency. Initial workup, including brain MRI and lumbar puncture, was unremarkable, but serum testing later revealed VGKC antibodies. Further oncological and gastrointestinal workup ruled out underlying malignancies or metabolic causes, including vitamin deficiencies and cirrhosis-related encephalopathy. The patient was treated with monthly intravenous immunoglobulin (IVIG) therapy and seizure prophylaxis with levetiracetam (Keppra), and continues to follow up for management of neurological symptoms and monitoring for potential disease recurrence.

### DISCUSSION

Diagnosing the etiology of Limbic Encephalitis can be challenging, particularly in the absence of an underlying malignancy. Anti-VGKC Encephalitis is an autoimmune condition that predominantly affects males over the age of 65 and is a recognized cause of Limbic Encephalitis. One third of patients can also have one or more movement disorders including ataxia, myoclonus, and tremors. While anti-VGKC encephalitis can occur as a paraneoplastic syndrome, particularly with thymoma and small cell lung cancer, most patients do not have an underlying malignancy.

### CONCLUSION

Anti-VGKC Antibody associated Limbic Encephalitis is diagnostically complex, especially in the absence of a known malignancy. High clinical suspicion is essential, and the presence of behavioral changes, cognitive impairment, and new-onset seizures should prompt consideration of autoimmune encephalitis in older adults. Timely diagnosis and initiation of immunotherapy can lead to significant clinical improvement.

